# The effect of tactile cueing on dual task performance in Parkinson’s disease. A systematic review and meta-analysis

**DOI:** 10.1016/j.prdoa.2024.100284

**Published:** 2024-11-17

**Authors:** Viktoria Azoidou, Alastair J Noyce, Cristina Simonet

**Affiliations:** a Centre for Preventive Neurology, Wolfson Institute of Population Health, Queen Mary University of London, London, UK; b Department of Neurology, Royal London Hospital, Barts Health, London, UK; c Brying Assessment and Rehabilitation Unit, Homerton University Hospital National Health Service Trust, London, UK

**Keywords:** Parkinson’s disease, Cues, Multitasking behaviour, Systematic review, Meta-analysis

## Abstract

**Background:**

Dual-task (DT) performance is impaired in Parkinson’s disease (PD), contributing to bradykinesia, postural instability, freezing of gait, and falls. Tactile cueing, including vibrotactile stimulation, has been suggested to improve DT performance in PD.

**Research Question:**

Does tactile cueing affect DT performance in PD, specifically measured by dual-task cost (DTC)?

**Methods:**

A systematic review was conducted in PubMed and EMBASE up to October 30, 2023, following the Preferred Reporting Items for Systematic reviews and Meta-Analyses (PRISMA) guidelines. Eligible studies were those in English that examined the effects of tactile cueing and/or closed-loop vibrotactile stimulation on DT performance in adults over 18 with idiopathic PD. The primary outcome, DTC, was calculated as the percentage change in performance from DT to single-task using the formula: [(single-task − dual-task)/single-task]* 100. A *meta*-analysis using a random-effects model pooled standardized mean differences (SMD) of DTC. Statistical significance was set at p < 0.05.

**Results:**

From 130 initially identified studies, eight were included in the review. Four studies with 374 participants were included in *meta*-analyses focusing on walking speed and step length. Three of the four studies indicated that tactile cueing improved DTC for these parameters. However, the SMD for walking speed (−109.69; 95 % CI −454.89 to 235.51, p = 0.39) and step length (−14.21; 95 %CI −53.25 to 24.83, p = 0.33) showed weak evidence of improvement.

**Conclusion:**

The *meta*-analysis provides weak evidence that tactile cueing may enhance walking speed and step length in DT conditions in PD. Rigorous objective studies are still lacking in this field of research.

## Highlights

• Eight out of 130 studies were included for systematic review.
• Four studies (total of 374 participants) were selected for *meta*-analysis on walking speed and step length.
• The *meta*-analysis showed weak evidence that tactile cueing improves walking speed or step length.
• The results of *meta*-analyses were affected by the limited number of studies.
• There is the need for standardized protocols in this research area of Parkinson’s.

## 1 Introduction

Dual-task (DT) performance refers to the ability to simultaneously manage and execute two distinct tasks, requiring the allocation of attention and cognitive resources to both activities [1]. The decrease in performance of one or both tasks in DT conditions is known as DT interference or DT cost (DTC) [2]. It is commonly seen that patients with Parkinson’s disease (PwP) experience motor and cognitive impairment, making it challenging for them to efficiently handle DT [3], [4], [5]. PwP require additional cognitive resources to compensate for dysfunction in the basal ganglia during DT performance. The challenges faced by PwP in DT are likely attributed to issues such as limited attention, deficits in central executive function, and a reduced level of task automatization [3]. Decreased performance on DT and greater DTC have consistently been linked to motor issues like bradykinesia, freezing of gait (FOG), postural instability, and falls [4], [5]. In DT conditions, unlike healthy adults who can demonstrate the ability to prioritize postural stability over secondary tasks, PwP are more likely to deviate from ‘posture first’ strategy [6]. The ‘posture first’ strategy highlights the significance of preserving postural stability to prevent falls and related injuries in challenging environments such as DT [6], [7].

While first line pharmacological therapies such as levodopa effectively address motor symptoms in PwP, they provide limited relief for gait issues, FOG, and DT difficulties [8]. They may also carry side effects such as impulse control disorders for dopamine agonists and wearing off periods and dyskinesias for levodopa [9], [10]. Moreover, in the advanced stage of PD, therapeutic interventions include device-aided therapies such as continuous subcutaneous apomorphine infusion (CSAI), levodopa-carbidopa intestinal gel (LCIG) infusion, and deep brain stimulation (DBS) [11]. Such device aided therapies, while more effective in some situations, require careful consideration due to adverse events, limited suitability, high cost, and invasiveness [11], [12]. Guidelines to identify candidates for device-aided therapies are currently lacking [11]. They may also require careful consideration due to different countries (continents) and related health-systems organizations [11]. Although, non-invasive devices that deliver cueing may be less effective than invasive ones, they are easier to use, and potentially more feasible in wider population as the risk of complication is lower [12].

In the recent years, several non-invasive devices have been manufactured, and some of them demonstrate promise in improving motor performance and attention allocation and may provide targeted support for specific DT challenges [13]. However, auditory, and visual cues, while effective in addressing FOG and improving gait parameters in PwP [14], limit real-world practicality [13], [15]. In contrast, tactile cues including vibrotactile stimulation, specifically in closed-loop configurations, delivered through non-invasive devices such as CUE1 [16], tactile anklets [17], VibroGait [18], and Gondola [19] have shown promise in alleviating motor symptoms, improving gait parameters, and addressing FOG in PwP without the limitations of the visual and auditory cueing systems [13], [20], [21]. Nonetheless, there is limited knowledge regarding the effect of these modalities on DT performance and DTC in PwP.

This study provides a comprehensive review of various tactile cues and their effect on DT performance in PwP. The primary objective is to examine the effect of tactile cueing, in particular closed-loop vibrotactile stimulation, on DTC in PwP. Secondary objective is to compare the performance of the single task (ST) versus the motor task under DT conditions.

## 2 Methods

### 2.1 Literature search

A systematic literature search of PubMed and EMBASE was performed for studies published until October 30, 2023, using the following keywords: Parkinson’s disease, cues, and multitasking behaviour. The reporting in systematic review and *meta*-analysis was done based on the Preferred Reporting Items for Systematic reviews and Meta-Analyses (PRISMA) guidelines [22]. The detailed search strategies for PubMed MeSH search are provided in the Supplementary Material 1.

### 2.2 Eligibility criteria

Studies were considered eligible for inclusion if they were 1) in the English language; 2) investigated the effects of tactile cueing and/or closed-loop vibrotactile stimulation on DT performance in people with idiopathic Parkinson’s disease (PD) over 18 years of age. Review of reference lists of the related articles and screening for potentially relevant conference abstracts were performed. The exclusion criteria included studies that: 1) were literature or systematic reviews, case reports, letters to editors, editorials, comments, conference abstracts, or book chapter; 2) included animal or cell models; 3) included people with concurrent invasive (deep brain) stimulation; 4) lacked appropriate outcome measures; 5) were in a form of protocols for randomized controlled trials (RCTs); 6) did not have quantitative data; 7) had no full text available; 8) investigated the effect of other types of cueing (e.g., auditory, visual, attentional) other than tactile; 9) achieved less than 7 out of 11 on the Critical Appraisal Skills Programme (CASP) criteria assessing study quality [23]. Regarding duplicate publications reporting the same cohort, the study with the largest sample size was included.

### 2.3 Data extraction

Two reviewers (V.A. and C.S.) independently extracted the following data from included studies: 1) study characteristics (the first author, publication year, study design); 2) population parameters (sample size, age, gender); 3) assessment of PD (scale of diagnosis, disease duration, clinical parameter values/outcomes); 4) intervention involving tactile cueing/ vibrotactile stimulation (experimental procedure, medication phase of participants at the time of assessment with tactile cueing, equipment and stimulation settings used for delivering tactile cueing); 5) inclusion of a sham/placebo device; 6) assessment of DT (type of primary and secondary task); 7) DTC which is the relative change in performance associated with DT expressed as a percentage (%) of each individual's ST performance and calculated based on the equation [single task-dual task]/single task*100] [2]; 8) mean (M) and standard deviations (SD) and their corresponding p values.

### 2.4 Quality assessment

The methodological quality of all eligible studies in the systematic review was independently assessed by V.A. and C.S. If discrepancies were found between the reviewers, a third reviewer (A.N.) would provide an independent opinion.

The relevant CASP checklist [23] form was used, according to the study design, to assess the quality of the studies (RCTs, case-control studies etc). Studies were included if each achieved a score of 7 or more.

### 2.5 Statistical analysis

The DTC was calculated to assess the percentage change in the primary task performance due to DT condition, separately, for each outcome, with cueing versus non-cueing intervention, using the equation: [single task-dual task]/single task*100] [2], unless the values were already available in the included studies. In this analysis, the M ± SD of the outcome measures in both ST and DT conditions were retrieved from publications, with and without tactile cueing or vibrotactile stimulation, for the entire sample of PwP in each study. In cases where data for the entire cohort were unavailable, we derived the values by summing the M and SD scores separately for sub-groups of PwP and, then, dividing the total scores by the respective sub-group number for each study.

A quantitative *meta*-analysis of the studies was included in the systematic review to merge results for the outcomes reported with the same outcomes in more than two studies. IBM SPSS version 28 (IBM Corp, Armonk, New York, United States of America) was used to conduct the *meta*-analysis. The outcomes selected for the *meta*-analysis were gait parameters, specifically, walking speed and step length under ST and DT conditions. These were the parameters where sufficient data was available, to allow for calculation of effect sizes (ES) and standard mean differences (SMD), along with their corresponding 95 % confidence intervals (CIs). Random effect models were used to pool ES as it was anticipated that there will be a significant between-study heterogeneity. As there was a small number of studies and the outcome measures were continuous variables, the restricted maximum likelihood estimator was used to determine the variance of the distribution of the true ES. Knapp Hartung adjustment [24] was used to calculate the CI around the pooled effect. ES were calculated between the M of patients’ parameters for ST and DT conditions, and the DTC pre-intervention and immediately post-intervention, as all studies assessed the acute effect of the intervention. Forest plots were created to summarise information on individual studies and heterogeneity and show the estimated common effect. Contour-enhanced funnel plots were created to investigate publication and other biases in *meta*-analysis. Statistical significance was set at *p* < 0.05.

## 3 Results

### 3.1 Literature search

No discrepancies were found between the independent reviewers C.S. and V.A. during the assessment of the articles.

**Fig. 1:**
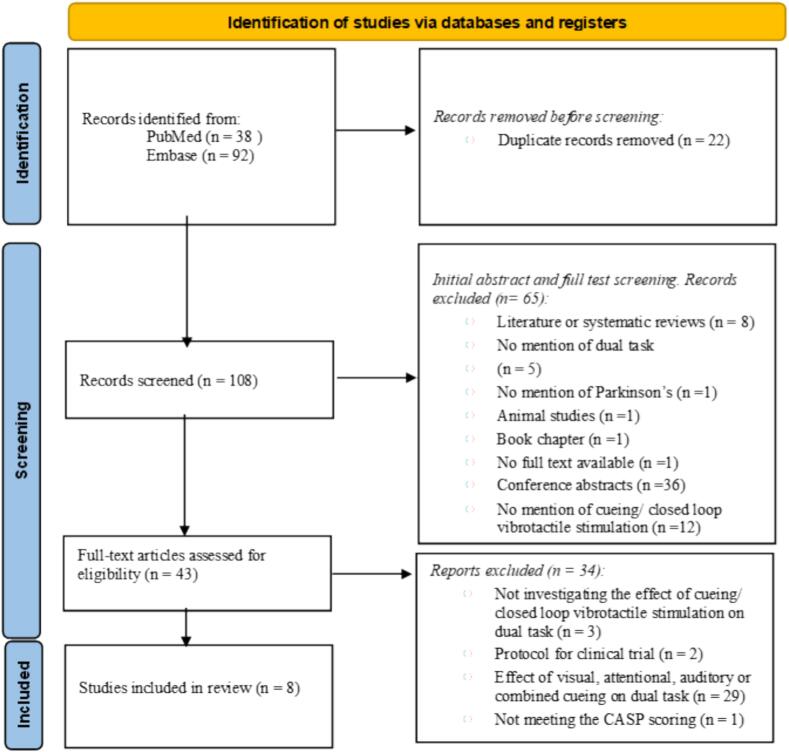
PRISMA flowchart illustrates study identification.

The literature search identified 130 articles, with no additional publications found in reference lists. Of the 43 articles assessed for eligibility, 29 were excluded as they primarily investigated the impact of visual, attentional, auditory, or combined cueing on DT performance. Nine articles met the initial inclusion criteria [18], [25], [26], [27], [28], [29], [30], [31], [42]. Eight articles [18], [25], [26], [27], [28], [29], [30], [31] met the final inclusion according to CASP criteria. These studies focused on examining the effects of tactile cueing and/or open- and closed-loop vibrotactile stimulation on DT performance. While some of the studies also investigated auditory and visual cueing, this review exclusively focused on the influence of tactile cueing and closed-loop vibrotactile stimulation on DT performance, excluding other types of cueing in the results section. The study identification process is illustrated in the PRISMA flow chart (Fig. 1).

### 3.2 Study characteristics and quality assessment

The systematic review presents a summary of eight studies, as outlined in Table 1. Information including study and year, study design, participant groups, sample size, demographic characteristics (i.e., age, sex, disease severity assessed with Hoehn & Yahr scale, and disease duration), as well as scores from PD-related questionnaires are included. The M average scores achieved on the cognitive function screening tools included in the studies, demonstrated that PwP had intact cognitive function. However, some PwP had poorer cognitive function as observed from the SD scores. Some studies also included healthy controls [27] or assessed PwP in sub-groups of with and without episodes of FOG [18], [29], [30] or postural misalignment [31]. No serious adverse events or reactions to the intervention were reported [18], [25], [26], [27], [28], [29], [30], [31].

**Table 1:** Characteristics of the included studies in the systematic review.

Study	Year	Study design	Groups & sample size	Age (mean ± SD years)	Sex (M/ F)	PD severity (H &Y) & duration (mean ± SD years)	PD related questionnaires (mean ± SD)	Cognitive assessment score (mean ± SD)
Rochester et al. [21]	2007	Cross-sectional	PD: 153	67.06 ± 7.54	M: 88 F: 65	H & Y: 2–4 Duration: 8.25 ± 5.09	UPDRS (total score): 56.03 ± 16.01 FOGQ: 8.73 ± 5.09	MMSE: 28.17 ± 1.82
Rochester et al. [22]	2010	RCT	PD (eERC): 76 PD (IERC): 77	eERC: 67.5 IERC: 69 SD not given.	eERC: M: 48 F: 28 lERC: M: 40 F: 37	H & Y: 2.5–3 Duration: eERC: 7 lERC: 8 SD not given.	UPDRS Part III: eERC: 31 lERC: 34 SD not given**.**	MMSE: eERC: 28.5 lERC: 29 SD not given.
Ivkovic et al. [23]	2016	Case controlled study	HC: 10 PD: 10	HC: 69 ± 7.4 PD: 71 ± 8.2	HC: M: 6F: 4 PD: M: 6F: 4	H & Y: 2.8 ± 0.5 PD duration not given.	none	MMSE: PD: 29.0 ± 1.1 HC: 30.0 ± 0.0
Mancini et al. [17]	2018	Case controlled study	PD + FOG: 25 PD-FOG: 18	PD + FOG: 69 ± 7 PD-FOG: 70 ± 7	PD + FOG: M: 19F: 6 PD-FOG: M: 14F: 4	H & Y: 2–4 Duration: PD + FOG: 9.3 ± 6.5 PD-FOG: 8.2 ± 4.7	MDS-UPDRS Part III: PD + FOG: 47.1 ± 10.1 PD-FOG: 43.6 ± 11.6	MoCA: PD + FOG: 25.1 ± 4.6 PD-FOG: 26.2 ± 3.4
Fino & Mancini [24]	2020	Case controlled study	PD: 43	70 ± 7.3	M: 33 F:10	H & Y not given Duration: 8.6 ± 5.6	MDS-UPDRS Part III: 46 ± 11NFOG (n = 22)	MoCA: 25 ± 4.5
Stuart & Mancini [26]	2020	Pilot study	PD + FOG: 13 PD-FOG: 12	PD + FOG: 69.69 ± 4.21 PD-FOG: 68.67 ± 3.85	PD + FOG: M: 8F: 5 PD-FOG: M: 9F: 3	H & Y: 1–3 PD + FOG: 12.08 ± 6.18) PD-FOG: 7.75 ± 5.77	UPDRS Part III: PD + FOG: 40.00 ± 14.01 PD-FOG: 32.50 ± 7.15 FOGQ: PD + FOG: 13.69 ± 6.89 PD-FOG: 0	MoCA: PD + FOG: 26.08 ± 3.62 PD-FOG: 28.25 ± 3.62
Schlenstedt et al. [25]	2020	Pilot study	PD + FOG: 18 PD-FOG: 18	PD + FOG: 67.8 ± 5.2 PD-FOG: 70.3 ± 7.0	PD + FOG: M: 14F: 4 PD-FOG: M: 14F: 4	H & Y: PD + FOG: 2.4 ± 0.6 PD-FOG: 2.1 ± 0.5 Duration: PD + FOG: 9.1 ± 6.6 PD-FOG: 8.2 ± 4.7	MDS-UPDRS Part III: PD + FOG: 45.3 ± 10.0 PD-FOG: 43.6 ± 11.6 NFOG: PD + FOG: 16.8 ± 6.7 PD-FOG: 0	MoCA: PD + FOG: 26.3 ± 3.5 PD-FOG: 26.2 ± 3.4
Stuart et al. [27]	2022	Pilot study	PD + PM: 10 PD-PM: 15	PD + PM: 68.9 ± 5.3 PD-PM: 70.2 ± 7.2	PD + PM: M: 9F: 1 PD-PM: M: 11F: 4	H & Y: 2–3 PD + PM: 16.4 ± 6.6 PD-PM: 7.9 ± 4.7	UPDRS Part III: PD + PM: 41.9 ± 12.1 PD-PM: 36.2 ± 9.7	MoCA: PD + PM: 26.3 ± 2.9 PD-PM: 27.1 ± 3.4

SD, Standard deviation; M, Male; F, Female; PD, Parkinson’s disease; H & Y, Hoehn & Yahr; UPDRS, United Parkinson’s Disease Rating Scale; FOGQ, Freezing of Gait Questionnaire; MMSE, Mini Mental State Exam; RCT, Randomised controlled trial; eERC, early External Rhythmical Cueing; lERC, late External Rhythmical Cueing; HC, Healthy Controls; FOG, Freezing of Gait; MDS-UPDRS, Movement Disorder Society-sponsored United Parkinson’s Disease Rating Scale; MoCA, Montreal Cognitive Assessment tool; NFOG, New Freezing of Gait Questionnaire; PM, Postural Misalignment.

The review prioritized high-quality studies regardless of their design. Notably, among the included studies, there was only one identified as an RCT [26]. Table 2a provides the quality assessment based on the CASP checklist for RCTs, while Table 2b summarises pilot and case studies.

**Table 2:** Quality assessment of the included studies based on the Critical Appraisal Skills Programme (CASP) checklist.

Study	**Year**	**Overall score**	Q1*	Q2*	Q3*	Q4*	Q5*	Q6*	Q7*	Q8*	Q9*	Q10*	Q11*
A. Quality assessment of randomised controlled trials based on CASP checklist for randomised controlled trials.													

Rochester et al. [22]	2010	7.5/11	Y	Y	CT	Y	Y	Y	Y	N	N	N	Y
Y, Yes; N, No; CT, Cannot Tell; *Q1: Did the study address a clearly focused research question? *Q2: Was the assignment of participants to interventions randomised? *Q3: Were all participants who entered the study accounted for at its conclusion? *Q4: Were the participants ‘blind’ to intervention they were given?, Were the investigators ‘blind’ to the intervention they were giving to participants?, Were the people assessing/analysing outcome/s ‘blinded’? *Q5: Were the study groups similar at the start of the randomised controlled trial? *Q6: Apart from the experimental intervention, did each study group receive the same level of care (that is, were they treated equally)? *Q7: Were the effects of intervention reported comprehensively? *Q8: Was the precision of the estimate of the intervention or treatment effect reported? *Q9: Do the benefits of the experimental intervention outweigh the harms and costs? *Q10: Can the results be applied to your local population/in your context? *Q11: Would the experimental intervention provide greater value to the people in your care than any of the existing interventions?													

B. Quality assessment of case control studies based on CASP checklist for case control studies.														
Study	**Year**	**Overall score**	Q1*	Q2*	Q3*	Q4*	Q5*	Q6 (a)*	Q6 (b)*	Q7*	Q8*	Q9*	Q10*	Q11*
Rochester et al. [21]	2007	9.5/11	Y	Y	Y	N	Y	Y	N	Y	Y	Y	Y	Y
Ivkovic et al. [23]	2016	10/11	Y	Y	Y	Y	Y	Y	Y	Y	Y	Y	N	Y
Mancini et al. [17]	2018	8.5/11	Y	Y	Y	CT	Y	N	Y	Y	Y	Y	N	Y
Fino & Mancini [24]	2020	9/11	Y	Y	Y	CT	Y	CT	N	Y	Y	Y	Y	Y
Schlenstedt et al. [25]	2020	9/11	Y	Y	Y	N	Y	Y	Y	Y	Y	Y	N	Y
Stuart & Mancini [26]	2020	8.5/11	Y	Y	Y	N	Y	CT	Y	Y	Y	Y	N	Y
Stuart et al. [27]	2022	7.5/11	Y	Y	Y	N	N	Y	N	Y	Y	Y	N	N

Y, Yes; N, No; CT, Cannot Tell; *Q1: Did the study address a clearly focused issue? *Q2: Did the authors use an appropriate method to answer their question? *Q3: Were the cases recruited in an acceptable way? *Q4: Were the controls selected in an acceptable way? *Q5: Was the exposure accurately measured to minimise bias? *Q6 (a): Aside from the experimental intervention, were the groups treated equally? *Q6 (b): Have the authors taken account of the potential confounding factors in the design and/or in their analysis? *Q7: How large was the treatment effect? *Q8: How precise was the estimate of the treatment effect? *Q9: Do you believe the results? *Q10: Can the results be applied to the local population? *Q11: Do the results of this study fit with other available evidence?

### 3.3 Summary of technologies delivering tactile cueing and/or vibrotactile stimulation

Table 3a shows features of technologies that provided tactile cueing and/or vibrotactile stimulation, detailing the name of the devices, their positioning on the body, and type of stimulation. Among the included studies, VibroGait [32] emerged as the predominant device, employed in four studies [18], [28], [29], [30]. The VibroGait is a wearable system that connects to the Opal positioned on the shins and comprises a controller unit (Arduino microcontroller). This controller unit utilizes a gyroscope to detect when the foot is in contact with the ground, triggering the tactor unit to produce vibrations, in case of the included studies [18], [28], [29], [30] to the wrist. The tactors utilize C-2 tactors from Engineering Acoustic, Inc., with a primary resonance falling within the 200–300 Hz range. The vibration intensity closely resembles that of a cell phone operating in vibration mode. None of the studies explored their intervention against a sham/placebo device.

**Table 3:** Study characteristics on a) medication phase, device, its position and stimulation, and if a sham/placebo device was used in the study, and b) single and dual tasks that were assessed in the studies as well as the outcome measures during dual task paradigms and their dual task costs with and without the tactile cueing device.

A. Medication phase, device and its position and stimulation.						
Study	**Year**	**Device**	**Position**	**Stimulation**	**Medication phase (ON/OFF)**	**Was the intervention assessed against sham/placebo device? (Yes/No)**
Rochester et al. [21]	2007	A prototype device	Waist	Delivered by a miniature cylinder worn under a wristband. Settings of stimulation not provided.	ON	No
Rochester et al. [22]	2010	A prototype device	Waist	Delivered by a miniature cylinder worn under a wristband. Settings of stimulation not provided.	ON	No
Ivkovic et al. [23]	2016	A 0.16 kg smartphone (MyTouch-3G™ HTC, Bellevue, Washington)	humero-radial joint	Vibrator oscillating at 100 Hz, consisted of 100 ms vibration pulses at the desired tactile cueing intervals.	ON	No
Mancini et al. [17]	2018	VibroGait	Wrist	The tactors are C-2 tactors (Engineering Acustic, Inc) with a primary resonance in the 200–300 Hz range. The vibration intensity is like that of a cell phone operating in vibration mode [28]	OFF	No
Fino & Mancini [24]	2020	VibroGait	Wrist	As for VibroGait from [28]	ON	No
Schlenstedt et al. [25]	2020	VibroGait	Wrist	As for VibroGait [28]	OFF	No
Stuart & Mancini [26]	2020	VibroGait	Wrist	As for VibroGait [28]	OFF	No
Stuart et al. [27]	2022	UpRight Go	Trunk	The device synched via Bluetooth with a mobile-phone application, with the sensor set to ‘training’ mode. The feedback signal consisted of a vibration at ∼ 100 Hz.	ON	No

B. Single and dual tasks that were assessed in the studies as well as the outcome measures during dual task paradigms and their dual task costs without and with the tactile cueing device. The dual task cost percentages (%) are shown for the full PD cohort in each study.							
Study	**Year**	**Single task/s (ST/STs)**	**Dual task/s (DT/DTs)**	**Outcome measures used during DT paradigms**	**Dual task cost (DTC) mean scores for outcome measures without tactile cueing**	**DTC mean scores for outcome measures with tactile cueing**	**DTC mean improvement-difference scores for outcome measures**
Rochester et al. [21]	2007	6 m walking, turn around through 180°	ST while carrying a tray with two glasses of water	Walking speed (m/s) Step length (m)Cadence (steps/min)	−12.60 % −12.73 % 0.38 %	5.95 % −6.99 % 1.26 %	−6.65 % −5.74 % 0.88 %
Rochester et al. [22]	2010	6 m walking	ST while carrying a tray with 2 glasses of water	Walking speed (m/s)Step length (m)Cadence (steps/min)	before training: 11.58 % after training: 8.82 % before training: 12.73 % after training: 10.17 % before training: −0.30 % after training: −0.27 %	before training: 7.61 % after training: 7.92 % before training: 9.10 % after training: 8.47 % before training: −0.21 % after training: −0.04 %	3.97 % 0.90 % 3.63 % 1.70 % −0.09 % −0.23 %
Ivkovic et al. [23]	2016	a) tapping heel b) straight line walking along a 150 m x 4 m hallway	ST while holding a tray with cups of water	Walking speed (m/s)Step length (m) Cadence (steps/min)	Not given	Not given	Not given
Mancini et al. [17]	2018	a) 2-minute walk on an 8-m walkway b) 1 min turning in place	ST while subtracting of 3′s from a 3-digit number	FOG ratio # of turns Average peak velocity (degrees/s)Average jerkiness (m^2^/s^5^)	14.99 % 16.69 % 17.66 % 21.11 %	14.52 % 13.54 % 12.48 % 14.35 %	0.47 % 3.15 % 5.18 % 6.76 %
Fino & Mancini [24]	2020	six, 2-minute walking trials between lines marked 7.6 m apart.	ST while reciting every other letter of the alphabet	Walking speed (m/s)Step length (m) Stride time (s)Step time (ms)Step Time SD (ms) Step Time Asymmetry (ms) Weight Transfer − λHS Early Swing – λTO Late Swing − λMS	12.50 % 7.53 % −6.48 % −5.72 % −13.64 % −86.67 % 16.67 % 0 % 0 %	8.11 % 6.98 % 0 % −0.16 % −14.81 % 25.93 % 27.27 % 0 % 0 %	4.39 % 0.55 % −6.48 % −5.56 % −1.17 % −60.74 % 11.40 % 0 % 0 %
Schlenstedt et al. [25]	2020	Walking for 2-minutes, back and forward on an 8 m walkway	ST while counting backwards by 3 from a 3-digit number	ML size of APA (g) AP size of APA (g)APA duration (s)Latency (s) 1st step range of motion1st step duration (s) TFL Co-contraction during APA (%)	8.03 % −1.19 % −16.89 % 26.69 % 6.27 % −11.96 % −54.56 %	3.26 % −4.41 % −13.80 % −13.89 % 11.46 % 7.90 % −29.64 %	4.77 % −3.22 % −3.09 % 12.80 % −5.19 % −4.06 % −24.92 %
Stuart & Mancini [26]	2020	9 m walking and turning	ST while performing the AX-CPT	Walking speed (m/s)Step length (m)Cadence (steps/min) Foot strike angle ⁰	−6.63 % −1.49 % 5.30 % −19.32 %	3.09 % 4.18 % 7.67 % 6.46 %	−3.54 % 2.69 % −2.37 % −12.86 %
Stuart et al. [27]	2022	2-minute of: a) Sitting on a high-stool with no back with eyes open, b) standing, feet at 10 cm apart, eyes open, looking at a poster painting, and c) walking at comfortable speed (back and forth over ∼ 10 m).	ST while performing the Welscher forward digit span	Neck (head sensor tilt) in standingTrunk (chest sensor tilt) in standingLower back (lumbar sensor tilt) in standing 2-minute angular displacement (neck flexion max) 2-minute angular displacement (lower back flexion max)	−19.80 % 15.20 % 0 % 11.91 % 22.67 %	24.83 % −1.92 % –22.72 % 0.39 % 32.59 %	5.03 % 17.12 % –22.72 % 11.52 % −9.92 %

kg, kilogram; m, metre; Hz, hertz; ms, milliseconds.

m, metre; s, second; min, minute; DTC was calculated using the equation [single task-dual task]/single task*100]; FOR, Freezing of Gait; m2, square metre; s2, squared second; ms, millisecond; λHS, divergence exponent λ; λTO, λ early swing; λMS, λ late swing; ML, medio-lateral; APA, anticipatory postural adjustment; g, gram; AX-CPT, Continuous Performance Task (e.g., participants listened to random letters and had to press a button held in their least Parkinson disease affected hand) every time the letter “I” followed an “A.”); AP, anterior-posterior; cm, centimetre; max, maximum.

### 3.4 Summary of DT paradigms and DTC

Table 3b demonstrates the characteristics of ST and DT assessments in the studies and the outcome measures observed in DT paradigms, both with and without the usage of a tactile cueing device. The focus of all studies was on ST and DT paradigms related to lower-limbs, falls, and FOG. None examined activities involving the upper-limbs. Walking was evaluated in seven studies. Five studies [25], [26], [27], [28], [29] assessed gait parameters including walking speed and step length. In DT conditions, three studies [25], [26], [27] incorporated a motor task as a secondary task. PwP performed better on ST than DT conditions in all studies with and without cueing effect [18], [25], [26], [27], [28], [29], [30], [31].

Closed-loop vibrotactile stimulation led to a significantly slower execution of the first step by increasing first step duration, and improved trunk stability [28] during walking under ST and DT condition [29]. Tactile cueing [25], [26], including closed-loop vibrotactile stimulation [30], also significantly improved walking speed and step length in ST and DT conditions, turning in PwP with FOG [18] and neck/upper back posture during sitting and standing [31].

Lower negative percentage DTC (i.e., better performance) was identified for specific outcome measures in some studies [28], [29], [30], [31], and for all outcome measures in others [18], [25], [26].The mean of DTC of all outcome measures across all studies ranged between −86.67 % to + 26.69 %, when the tasks were performed without cueing, and −29.64 % to + 32.59 %, when the same tasks were performed with cueing (Table 3b). Minimum improvement for DTC with cueing was 0 % [29] and maximum 60.74 % [28], while others [31] found that the DTC worsened (e.g., negatively increased) when cueing was applied during walking but improved during sitting and standing. One study [27] did not provide scores for DT and DTC for their outcomes by only reporting the result and p values.

### 3.5 Meta-analyses on the effect of cueing on walking performance

Meta-analysis was conducted only for walking speed and step length, as their M ± SD scores could be obtained from more than two studies. Specifically, four studies [25], [26], [28], [30] with a total of 374 participants were included in the analysis. The remaining four studies [18], [27], [29], [31] were excluded due to insufficient data, lacking values for M ± SD, and/or the absence of specific data on walking speed and step length scores before and after tactile cueing intervention.

**Fig. 2:**
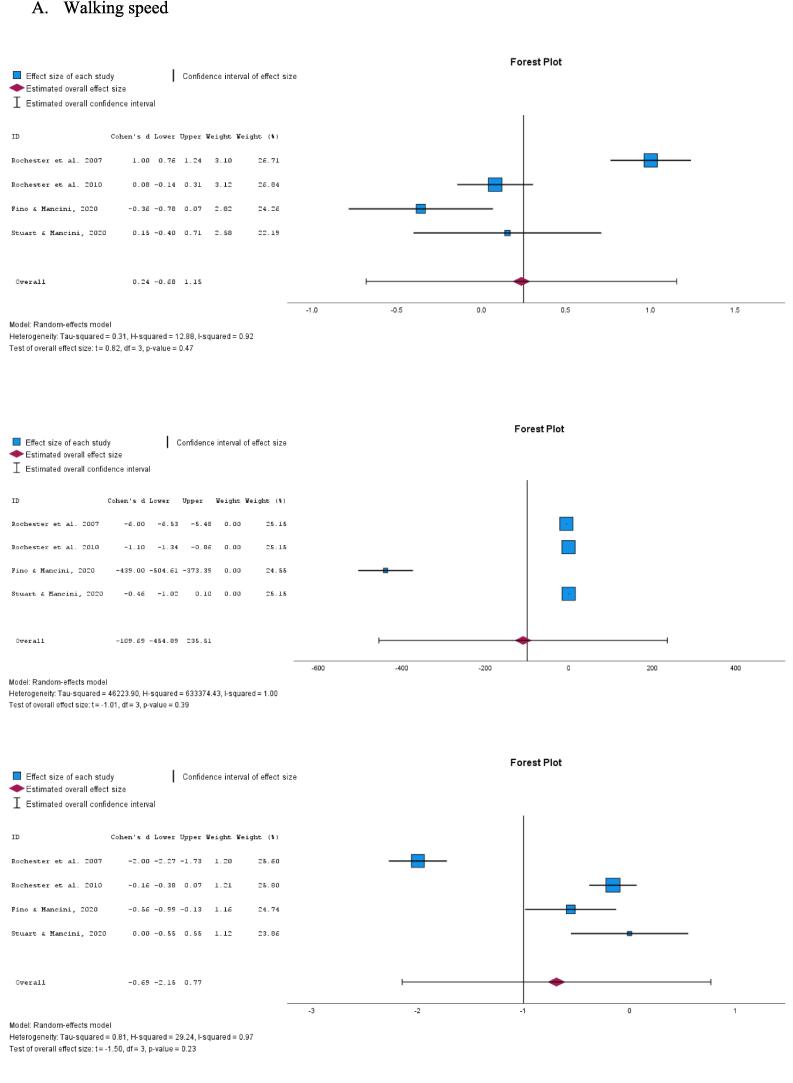

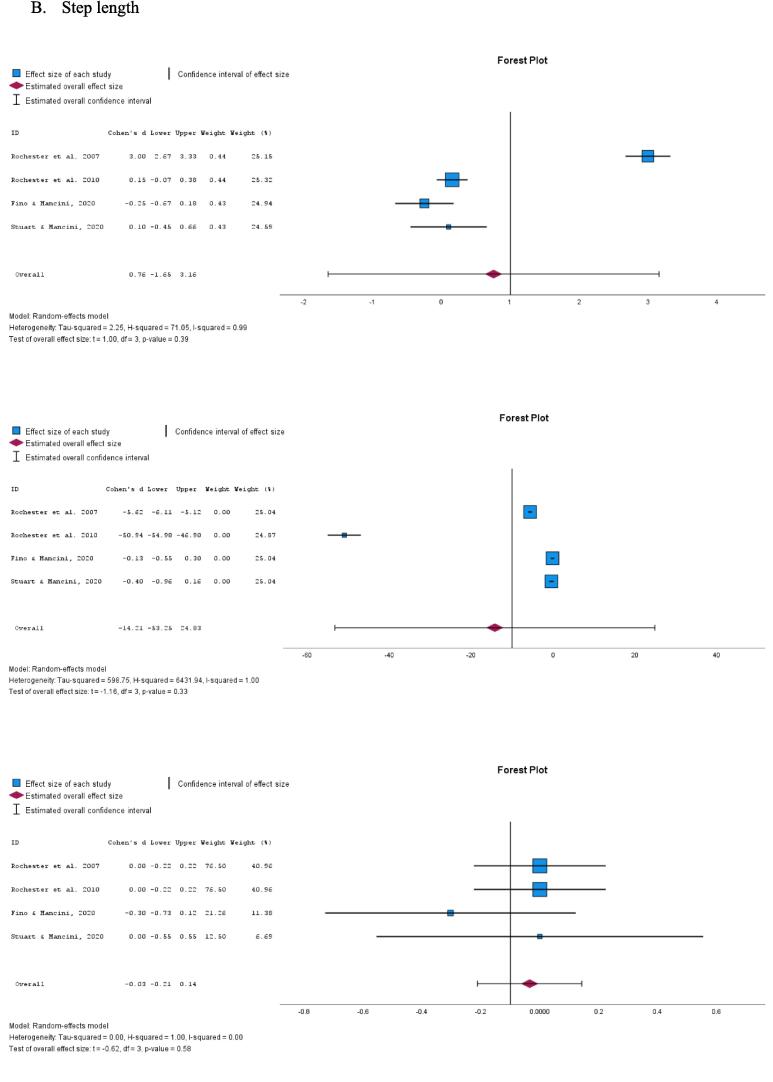
Forest plot of *meta*-analysis on the effect of tactile cueing on dual task, dual task cost and single task scores, respectively, for A) walking speed and B) step length.

The combined ES estimate across the four studies indicated a SMD value of 0.24 (95 % CI −0.68–1.15, p = 0.47, I^2^ = 92 %; Fig. 2A). This suggests weak evidence that the tactile cueing or closed-loop vibrotactile stimulation has a beneficial effect on walking speed under DT conditions compared to performing the task without cueing. Similarly, the combined ES estimate for DTC of walking speed was −109.69 (95 % CI −454.89–235.51, p = 0.39, I^2^ = 100 %; Fig. 2A), providing no convincing evidence that the tactile cueing improves the DTC of walking speed under DT conditions. Additionally, the pooled ES estimate for walking speed under ST conditions was −0.69 (95 % CI −2.15–0.77, p = 0.23, I^2^ = 97 %; Fig. 2A), indicating no convincing evidence of the beneficial effect of tactile cueing on walking speed.

In a parallel analysis, the combined ES estimate for step length under DT conditions was 0.76 (95 % CI −1.65–3.16, p = 0.39, I^2^ = 99 %; Fig. 2B), providing weak evidence on the beneficial effect of tactile cueing on step length compared to performing the same task without cueing. The pooled ES estimate for DTC of step length under DT conditions was −14.21 (95 % CI −53.25–24.83, p = 0.33, I^2^ = 100 %; Fig. 2B), indicating no convincing evidence on the beneficial effect of the tactile cueing on DTC for step length. Furthermore, the combined ES estimate for step length under ST conditions was −0.03 (95 % CI −0.21–0.14, p = 0.58, I^2^ = 0 %; Fig. 2B), demonstrating no convincing evidence on the beneficial effect of the tactile cueing on step length under ST conditions.

### 3.6 Publication bias assessment

Due to the limited number of studies included in the *meta*-analysis [25], [25], [28], [30], a contour-enhanced funnel plot for the ST and DT conditions as well as the DTC for walking speed and step length was performed to assess for publication bias. The assumption is that studies characterized by high precision cluster around the average, while studies with lower precision are distributed symmetrically on both sides of the average, forming a funnel-shaped distribution. Any deviation from this triangle share of the funnel plot may suggest the presence of publication bias. Funnel plot asymmetry was observed in all instances, except for step length under the ST condition (Supplementary Material 2, Figure A.i. walking speed; ii. step length). When asymmetry was detected in the funnel plot, it was not feasible to eliminate studies outside the hollow dots of the plot and re-conduct the *meta*-analysis. This was primarily because the number of studies, in such instances, were insufficient to re-run the *meta*-analysis.

## 4 Discussion

This review does not aim to duplicate previous studies that have assessed the effectiveness of tactile cueing, focused vibrotactile stimulation, or wearable non-invasive medical devices in PD [11], [12], [13], [20], [21]. It is well-established that poor DT performance is a significant risk factor for FOG, increased falls, and reduced independence, ultimately diminishing the overall quality of life in individuals with PD [4], [5]. Therefore, this review is the first one that specifically focuses on the impact of tactile cueing with vibrotactile stimulation on DT performance in PD.

Three articles demonstrated that using tactile cueing significantly reduced DTC for walking speed and step length compared to not using it. Although a *meta*-analysis of four studies showed weak evidence supporting the improvement of DTC for walking speed and step length with tactile cueing under ST and DT conditions, there are several reasons why the impact of tactile cueing on DT performance and DTC in PwP warrants further investigation, listed as follows.

Understanding the impact of tactile cueing on DT performance in PD is crucial for improving daily functioning [2]. DT assessments, which test motor and cognitive abilities together, offer insights into managing competing demands in PD [2]. Poor DT performance is linked to greater postural instability, higher fall risk, and FOG episodes [4], [5]. Cognitive issues, including mild cognitive impairment (25–30 % prevalence) [5], [33] and dementia (affecting 50 % within ten years of diagnosis) [34], worsen gait, balance, and DT performance, reducing independence and quality of life. Declines in executive functions, such as divided attention, working memory, and processing speed, further impair motor tasks and DT performance, affecting daily activities [3], [4], [5]. Comparative studies show PD patients have significantly worse DT performance than healthy controls [6], [27], and those with FOG [18], [29], [30]. Many studies include patients with low cognitive scores but lack subgroup analyses, highlighting the need for research on tactile cueing effects in PD patients with and without cognitive impairment [3]. Thus, findings may not generalize across all PD and cognitive impairment populations, underscoring the need for further research [3].

Understanding DT performance and finding effective strategies to improve it in PwP remains crucial. Most studies have focused on how secondary tasks affect primary tasks, but it is important to also understand how primary tasks influence secondary tasks for safety during walking [6], [7], [35]. PwP often prioritize secondary tasks over primary (motor) tasks, which can compromise balance and safety during daily activities [6], [7], [35]. Investigating whether tactile cueing can alter these prioritization strategies may improve safety. The best method for predicting DT interference in motor performance for this group is still unclear and requires more research. Despite comparing various secondary tasks may reveal differences in sensitivity [36], [37], using the DTC is recommended for assessing and comparing interference regardless of the secondary task [2]. A DTC over 10 % can significantly increase postural instability [38], while a DTC of 20 % or more in walking speed may raise the risk of falls [39]. Evaluating DTC in PwP can help identify those struggling with gait, balance, and daily tasks, and at higher risk of falls [36], [37].

Activities involving significant upper limb movement, such as reaching and carrying, can challenge balance and stability, heightening the fall risk in PwP. Surprisingly, this review found no studies on how tactile cueing affects upper-limb DT performance and DTC. A recent study [40] revealed that while lower-limb activities effectively differentiate PwP from controls, their effectiveness diminishes under DT conditions. However, DTC for arm range of motion proved more specific to PD and better distinguished PwP from healthy individuals during DT tasks like walking and reciting alternate letters of the alphabet [40].

Reviewed studies show that tactile cueing and vibrotactile stimulation improve DT performance in PwP during ST and DT activities at an individual study level. Four of them were from the same research group [18], [28], [30], [31]. However, *meta*-analysis showed no significant effect on DT performance metrics like walking speed and step length, likely due to the limited number of studies and their variability. Factors such as the limitations of current measures in capturing Parkinson's-specific DT impairments need consideration. Alternative measures like arm range of motion, foot strike angle, turning velocity, and duration [18], [40] might better reflect reduced automaticity in PD. Future research should examine the impact of tactile cueing on upper limbs, trunk, and lower back, which are vital for postural stability in PwP [28], [31]. Exploring these effects could help develop tailored rehabilitation strategies that address both motor and cognitive aspects.

In this review, 29 articles were excluded for focusing on cueing effects other than tactile cueing for improving DT performance. Tactile cueing, particularly closed-loop vibrotactile stimulation, is often more suitable for home and outdoor settings due to its non-distracting nature [14], [15], and shows promise in enhancing motor symptoms in PwP during DT. Advances in technology have produced tactile cueing devices like CUE1 [16] and tactile ankles [17], offering benefits for managing motor symptoms in PD without medication side effects [8], [9], [10] or invasive procedures like deep brain stimulation [11], [12]. This review found four studies using the VibroGait cueing device [18], [28], [29], [30], indicating a need to explore other tactile devices and evaluate their effectiveness in improving DT performance in PwP. Future research should compare various tactile devices to better assess their effectiveness.

Future research should also investigate the long-term impact of various tactile cueing devices on DT performance in PwP. This review identified only two studies [25], [26] examining short-term tactile cueing effects on DT performance, both showing benefits lasting up to three weeks [25]. The remaining studies [18], [27], [28], [29], [30], [31] focused on immediate effects. Issues with retention were observed, with immediate changes in walking speed and step length linked to rapid learning, while cued gait retention supported long-term learning consolidation [26]. Uncertainty remains about the effects of training type, stimulation duration, and other factors on retention. Hence, further high-quality studies are needed for definitive insights.

The key takeaway from this work is that research in this field is still in its early stages. The reviewed studies are limited in scope and vary in quality, which calls for careful interpretation of their findings. Consistent with previous reviews [13], [20], [21], this review also indicates that, although most of the devices assessed are deemed low risk, there is currently insufficient evidence to endorse any of them for extensive research or clinical use. None of the devices have been approved for clinical application yet. Although some studies evaluated devices like Gondola [19] and CUE1 [16], which have received FDA approval and CE marking, respectively, these devices were not included in this review. Despite showing evidence of increasing stride length and gait speed in PD patients [19], [43] and improvements in gait speed, motor symptoms, FOG, balance, and falls [16], they were excluded due to a lack of research specifically addressing their effects on DT performance in PD.

Comparing devices across studies was challenging due to compatibility issues, highlighting the need for standardized protocols for consistent application and comparison. The lack of sham control groups also limits result validity. Studies [44], [45], [46] have examined vibrotactile cueing against sham devices on gait; for instance, a crossover double-blind study found that Equistasi, targeting the 7th cervical vertebra and soleus tendons, improved walking velocity, stride length, and stance but increased double support percentage (left and right stride) compared to placebo [44]. Another sham-controlled study using Gondola™ showed that PwP maintained improved gait parameters for up to four weeks post-treatment [45], [46]. However, in the current review, none of the studies assessed the effect of vibrotactile cueing on dual-task performance or long-term outcomes, nor addressed device practicality for daily use.

The mechanisms behind tactile cueing responses in DT gait performance remain unclear. It is proposed that PwP may use more cortical attention resources during DT gait tasks than healthy controls [2], potentially impairing gait performance due to limited attention resources [35]. External cues, such as tactile cueing and vibrotactile stimulation, might shift gait control from automatic to voluntary, involving parieto-premotor pathways and providing an alternative to basal ganglia circuits in PwP [18], [30], [31].

Recent advancements in functional near-infrared spectroscopy and electroencephalography (EEG) allow for monitoring cortical activity during gait in PwP [41]. Studies utilizing these methods have indicated that closed-loop tactile cueing affects attentional processing and EEG frequencies [30]. Stuart & Mancini [30] found that tactile cueing did not significantly alter prefrontal cortex activity during walking or turning, suggesting it modifies walking behavior without impacting executive attention resources beyond those required for DT performance. However, the variability in cueing methods and task complexities underscores the need for a better understanding of cueing mechanisms to enhance rehabilitation [25]. Exploring how tactile cueing influences DT performance can help tailor treatments for PwP, integrating these techniques into therapy to improve motor and cognitive functions and advancing treatment approaches for PD.

This review has certain limitations. It only included English-language studies, potentially missing relevant research in other languages. The authors selected only two primary databases, PubMed and EMBASE, potentially excluding studies from others like Cochrane, which—though not featuring primary studies—provides valuable systematic reviews that could reveal additional relevant articles. Given the emerging nature and limited studies on tactile cueing and vibrotactile stimulation, future research should consider screening clinicaltrials.gov to identify ongoing trials in this area. Notably, the authors did not find any studies examining vibrotactile stimulation's impact on DT performance in PD. The *meta*-analysis was limited by the small number of articles. Although the included articles were of sufficient quality, their heterogeneity and lack of high-quality RCTs emphasize the need for more research and standardized protocols in the field.

## 5 Conclusion

The literature reveals considerable variability and a lack of comprehensive research on the effects of tactile cueing, including vibrotactile stimulation, on DT performance. Of the four studies reviewed, three suggest that tactile cueing, such as vibrotactile stimulation, may improve DT gait performance. However, the *meta*-analysis points to limited evidence regarding its impact on walking speed and step length in PwP. While non-invasive devices that provide cueing and/or vibrotactile stimulation are low risk and show potential for improving gait parameters in PD, the current evidence for their effectiveness is relatively weak. More rigorous research is needed, particularly focusing on the long-term effects of tactile cueing on DT performance. Such studies could enhance management strategies, improve rehabilitation outcomes, reduce fall risk, and ultimately improve the quality of life for PwP.

## 6 Funding source and conflict of interest

This work was supported by Knowledge Transfer Partnership (KTP) United Kingdom 2021 to 2022, round 4, UKRI KTP (Innovate UK).

V.A is employed by Queen Mary University of London to work with Charco Neurotech Ltd but she is not employed by Charco Neurotech Ltd. V.A., C.S., and A.J.N. hold a grant (KTP UK 2021 to 2022, round 4, UKRI KTP Innovate UK) to evaluate the effectiveness of the CUE1 device in PwP. A.J.N. is an external advisor to Charco Neurotech Ltd. A.J.N. is Associate editor at Journal of Parkinson’s Disease but will not be involved anyhow in the process of reviewing this manuscript.

## 7 Financial disclosures for the previous 12 months

Prof Noyce reports grants from Parkinson's UK, Barts Charity, Cure Parkinson’s, National Institute for Health and Care Research, Innovate UK, Virginia Keiley benefaction, Solvemed, the Medical College of Saint Bartholomew’s Hospital Trust, Alchemab, Aligning Science Across Parkinson’s Global Parkinson’s Genetics Program (ASAP-GP2) and the Michael J Fox Foundation. Prof Noyce reports consultancy and personal fees from AstraZeneca, AbbVie, Profile, Bial, Charco Neurotech, Alchemab, Sosei Heptares, Umedeor and Britannia, outside the submitted work. Prof Noyce has share options in Umedeor. Prof Noyce is an Associate Editor for the Journal of Parkinson’s Disease.

Dr Cristina Simonet reports grants from Innovate UK and the Michael J Fox Foundation. She also works as a co-investigator with Roche, outside the submitted work.

## 8 Ethical compliance statement

The datasets generated during and/or analysed during the current study are available upon reasonable request from the corresponding author but also can be retrieved directly from the included studies as this is a systematic review and meta-analysis and did not include data collections from participants.

This systematic review and meta-analysis followed the PRISMA diagram. The quality assessment of articles was performed according to the Critical Appraisal Skills Programme (CASP) checklist.

Informed patient consent was not necessary for this work as this is a systematic review and meta-analysis and does not include actively involving human participants.

We confirm that we have read the Journal’s position on issues involved in ethical publication and affirm that this work is consistent with those guidelines.

## CRediT authorship contribution statement

**Viktoria Azoidou:** Writing – review & editing, Writing – original draft, Visualization, Software, Resources, Project administration, Methodology, Investigation, Formal analysis, Data curation, Conceptualization. **Alastair J Noyce:** Writing – review & editing, Supervision, Funding acquisition, Conceptualization. **Cristina Simonet:** Writing – review & editing, Supervision, Funding acquisition, Conceptualization.

## Declaration of competing interest

The authors declare that they have no known competing financial interests or personal relationships that could have appeared to influence the work reported in this paper.

## References

[1] Woollacott M., Shumway-Cook A. (2002). Attention and the control of posture and gait: a review of an emerging area of research. Gait Posture.

[2] Plummer P., Eskes G. (2015). Measuring treatment effects on dual-task performance: a framework for research and clinical practice. Front. Hum. Neurosci..

[3] Kelly V.E., Eusterbrock A.J., Shumway-Cook A. (2012). A review of dual-task walking deficits in people with Parkinson's disease: motor and cognitive contributions, mechanisms, and clinical implications. Parkinson's Disease.

[4] Heinzel S., Maechtel M., Hasmann S.E., Hobert M.A., Heger T., Berg D. (2016). Motor dual-tasking deficits predict falls in Parkinson's disease: A prospective study. Parkinsonism Related Disorders.

[5] Onder H., Ozyurek O. (2021). The impact of distinct cognitive dual-tasks on gait in Parkinson's disease and the associations with the clinical features of Parkinson's disease. Neurol. Sci.: Off. J. 0Italian Neurol. Soc. Italian Soc. Clin. Neurophysiol..

[6] Lin Y.P., Lin I.I., Chiou W.D., Chang H.C., Chen R.S., Lu C.S. (2023). The Executive-Function-Related Cognitive-Motor Dual Task Walking Performance and Task Prioritizing Effect on People with Parkinson's Disease. Healthcare (basel, Switzerland).

[7] Johansson H., Ekman U., Rennie L., Peterson D.S., Leavy B., Franzén E. (2021). Dual-Task Effects During a Motor-Cognitive Task in Parkinson's Disease: Patterns of Prioritization and the Influence of Cognitive Status. Neuro Rehab. Neural Repair.

[8] Smulders K., Dale M.L., Carlson-Kuhta P., Nutt J.G., Horak F.B. (2016). Pharmacological treatment in Parkinson's disease: Effects on gait. Parkinsonism Relat. Disord..

[9] Fahn S. (2006). A new look at levodopa based on the ELLDOPA study. J. Neural Transm. Suppl..

[10] Weiss H.D., Marsh L. (2012). Impulse control disorders and compulsive behaviors associated with dopaminergic therapies in Parkinson disease. Neurol. Clin. Pract..

[11] Marsili L., Bologna M., Miyasaki J.M., Colosimo C. (2021). Device-aided therapies for advanced Parkinson disease: insights from an international survey. Neurol. Sci.: Off. J. Ital. Neurol. Soc. of the Ital. Soc. Clin. Neurophysiol..

[12] Fujikawa J., Morigaki R., Yamamoto N., Oda T., Nakanishi H., Izumi Y. (2022). Therapeutic Devices for Motor Symptoms in Parkinson's Disease: Current Progress and a Systematic Review of Recent Randomized Controlled Trials. Front. Aging Neurosci..

[13] Cosentino C., Putzolu M., Mezzarobba S., Cecchella M., Innocenti T., Bonassi G., Botta A., Lagravinese G., Avanzino L., Pelosin E. (2023). One cue does not fit all: A systematic review with meta-analysis of the effectiveness of cueing on freezing of gait in Parkinson's disease. Neurosci. Biobehav. Rev..

[14] Ginis P., Nackaerts E., Nieuwboer A., Heremans E. (2018). Cueing for people with Parkinson's disease with freezing of gait: A narrative review of the state-of-the-art and novel perspectives. Ann. Phys. Rehabil. Med..

[15] Janssen S., Bolte B., Nonnekes J., Bittner M., Bloem B.R., Heida T. (2017). Usability of Three-dimensional Augmented Visual Cues Delivered by Smart Glasses on (Freezing of) Gait in Parkinson's Disease. Front. Neurol..

[16] Tan X.S., Pierres F., Dallman-Porter A., Hardie-Brown W., Kwon K.Y. (2021). Focused Vibrotactile Stimulation with Cueing Effect on Freezing of Gait in Parkinson's Disease: Two Case Reports. J. Movement Disord..

[17] Rossi S., Lisini Baldi T., Aggravi M., Ulivelli M., Cioncoloni D., Niccolini V. (2020). Wearable haptic anklets for gait and freezing improvement in Parkinson's disease: a proof-of-concept study. Neurol. Sci.: Off. J. Ital. Neurol. Soc. Ital. Soc. Clin. Neurophysiol..

[18] Mancini M., Smulders K., Harker G., Stuart S., Nutt J.G. (2018). Assessment of the ability of open- and closed-loop cueing to improve turning and freezing in people with Parkinson's disease. Sci. Rep..

[19] Stocchi F., Sale P., Kleiner A.F., Casali M., Cimolin V., de Pandis F., Albertini G., Galli M. (2015). Long-term effects of automated mechanical peripheral stimulation on gait patterns of patients with Parkinson's disease. International journal of rehabilitation research. Internationale Zeitschrift Fur Rehabilitationsforschung. Revue Internationale De Recherches De Readaptation.

[20] Sweeney D., Quinlan L.R., Browne P., Richardson M., Meskell P., ÓLaighin G. (2019). A Technological Review of Wearable Cueing Devices Addressing Freezing of Gait in Parkinson's Disease. Sensors (Basel, Switzerland).

[21] Muthukrishnan N., Abbas J.J., Shill H.A., Krishnamurthi N. (2019). Cueing Paradigms to Improve Gait and Posture in Parkinson's Disease: A Narrative Review. Sensors (Basel, Switzerland).

[22] Page M.J., McKenzie J.E., Bossuyt P.M., Boutron I., Hoffmann T.C., Mulrow C.D. (2021). The PRISMA 2020 statement: an updated guideline for reporting systematic reviews. BMJ (Clin. Res. Ed.).

[23] Critical Appraisal Skills Programme (2018). CASP (insert name of checklist i.e. Qualitative) Checklist. [online] Available at: https://casp-uk.net/casp-tools-checklists/. Accessed: 1 Jan 2024.

[24] Knapp G., Hartung J. (2003). Improved tests for a random effects meta-regression with a single covariate. Stat. Med..

[25] Rochester, L., Nieuwboer, A., Baker, K., Hetherington, V., Willems, A. M., Chavret, F., Kwakkel, G., et al. (2007). The attentional cost of external rhythmical cues and their impact on gait in Parkinson's disease: effect of cue modality and task complexity. Journal of neural transmission (Vienna, Austria : 1996), 114(10), 1243–1248. https://doi.org/10.1007/s00702-007-0756-y. 10.1007/s00702-007-0756-y 17598068

[26] Rochester L., Baker K., Hetherington V., Jones D., Willems A.M., Kwakkel G. (2010). Evidence for motor learning in Parkinson's disease: acquisition, automaticity and retention of cued gait performance after training with external rhythmical cues. Brain Res..

[27] Ivkovic V., Fisher S., Paloski W.H. (2016). Smartphone-based tactile cueing improves motor performance in Parkinson's disease. Parkinsonism Relat Disord.

[28] Fino P.C., Mancini M. (2020). Phase-Dependent Effects of Closed-Loop Tactile Feedback on Gait Stability in Parkinson's Disease. IEEE Trans. Neural Syst. Rehab. Eng.: Publ. IEEE Eng. Med. Biol. Soc..

[29] Schlenstedt C., Peterson D.S., Mancini M. (2020). The effect of tactile feedback on gait initiation in people with Parkinson's disease: A pilot study. Gait Posture.

[30] Stuart S., Mancini M. (2020). Prefrontal Cortical Activation With Open and Closed-Loop Tactile Cueing When Walking and Turning in Parkinson Disease: A Pilot Study. J. Neurol. Phys. Therapy : JNPT.

[31] Stuart S., Godfrey A., Mancini M. (2022). Staying UpRight in Parkinson's disease: A pilot study of a novel wearable postural intervention. Gait Posture.

[32] Harrington W. (2016). 2016 38th Annual International Conference of the IEEE Engineering in Medicine and Biology Society (EMBC).

[33] Svenningsson P., Westman E., Ballard C., Aarsland D. (2012). Cognitive impairment in patients with Parkinson's disease: diagnosis, biomarkers, and treatment. Lancet Neurol..

[34] Williams-Gray C.H., Mason S.L., Evans J.R., Foltynie T., Brayne C., Robbins T.W. (2013). The CamPaIGN study of Parkinson's disease: 10-year outlook in an incident population-based cohort. J. Neurol. Neurosurg. Psychiatry.

[35] Yogev G., Giladi N., Peretz C., Springer S., Simon E.S., Hausdorff J.M. (2005). Dual tasking, gait rhythmicity, and Parkinson's disease: which aspects of gait are attention demanding?. Eur. J. Neurosci..

[36] Zirek E., Ersoz Huseyinsinoglu B., Tufekcioglu Z., Bilgic B., Hanagasi H. (2018). Which cognitive dual-task walking causes most interference on the Timed Up and Go test in Parkinson's disease: a controlled study. Neurol. Sci.: Off. J. Ital. Neurol. Soc. Ital. Soc. Clin. Neurophysiol..

[37] De Freitas, T. B., MS, PT, Leite, P. H. W., BS, Doná, F., PhD, PT, Pompeu, J. E., PhD, PT, Swarowsky, A., PhD, PT, & Torriani-Pasin, C., PhD, PT (2020). The effects of dual task gait and balance training in Parkinson's disease: a systematic review. Physiotherapy Theory Practice, 36(10), 1088–1096. https://doi.org/10.1080/09593985.2018.1551455. 10.1080/09593985.2018.1551455 30501424

[38] Muir-Hunter S.W., Wittwer J.E. (2016). Dual-task testing to predict falls in community-dwelling older adults: a systematic review. Physiotherapy.

[39] Hollman J.H., Kovash F.M., Kubik J.J., Linbo R.A. (2007). Age-related differences in spatiotemporal markers of gait stability during dual task walking. Gait Posture.

[40] Vitorio R., Hasegawa N., Carlson-Kuhta P., Nutt J.G., Horak F.B., Mancini M. (2021). Dual-Task Costs of Quantitative Gait Parameters While Walking and Turning in People with Parkinson's Disease: Beyond Gait Speed. J. Parkinson's Dis..

[41] Stuart S., Vitorio R., Morris R., Martini D.N., Fino P.C., Mancini M. (2018). Cortical activity during walking and balance tasks in older adults and in people with Parkinson's disease: A structured review. Maturitas.

[42] Deepa S., Chitra M. (2015). Efficacy of external cueing on kinematic gait parameters during a dual motor task in Parkinson's disease. International Journal of Pharmaceutical Sciences Review and Research..

[43] Galli M., Vicidomini C., Rozin Kleiner A.F., Vacca L., Cimolin V., Condoluci C., Stocchi F., De Pandis M.F. (2018). Peripheral neurostimulation breaks the shuffling steps patterns in Parkinsonian gait: a double blind randomized longitudinal study with automated mechanical peripheral stimulation. Eur. J. Phys. Rehabil. Med..

[44] Peppe A., Paravati S., Baldassarre M.G., Bakdounes L., Spolaor F., Guiotto A., Pavan D., Sawacha Z., Bottino S., Clerici D., Cau N., Mauro A., Albani G., Avenali M., Sandrini G., Tassorelli C., Volpe D. (2019). Proprioceptive Focal Stimulation (Equistasi®) May Improve the Quality of Gait in Middle-Moderate Parkinson's Disease Patients. Double-Blind, Double-Dummy, Randomized, Crossover, Italian Multicentric Study. Front. Neurol..

[45] Pagnussat A.S., Salazar A.P., Pinto C., Redivo Marchese R., Rieder C.R.M., Alves Filho J.O., Franco A.R., Kleiner A.F.R. (2020). Plantar stimulation alters brain connectivity in idiopathic Parkinson's disease. Acta Neurol. Scand..

[46] Brognara L., Navarro-Flores E., Iachemet L., Serra-Catalá N., Cauli O. (2020). Beneficial Effect of Foot Plantar Stimulation in Gait Parameters in Individuals with Parkinson's Disease. Brain Sci..

