# Supplementary material for: The effect of tactile cueing on dual task performance in Parkinson’s disease. A systematic review and meta-analysis
Source: Clin Park Relat Disord. 2024 Nov 17;11:100284. doi: 10.1016/j.prdoa.2024.100284 (PMC11617393; doi:10.1016/j.prdoa.2024.100284)
Supplement: Supplementary Data 1 [file mmc1.docx]

# **SUPPLEMENTARY MATERIAL**

## **MeSH search strategies for PubMed**

[Cues OR cueing OR "Feedback, Sensory" OR "focused vibrotactile stimulation" OR "vibrotactile feedback" OR vibration OR "tactile stimulation"] AND ["Multitasking Behavior" OR "dual task" OR "concurrent tasks" OR "dual task training" OR "cognitive motor interference" OR "dual task cost" OR "divided attention"] AND ["Parkinson Disease"].

## **Figures**

1. Walking speed


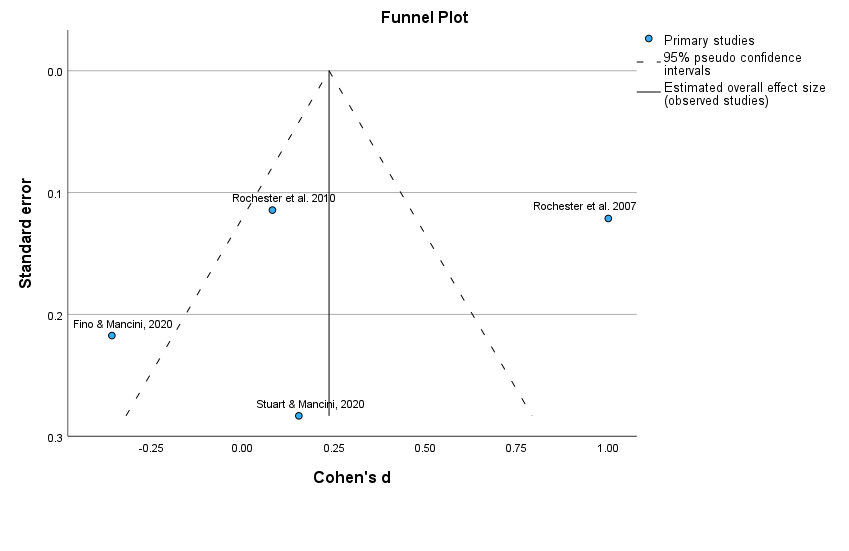


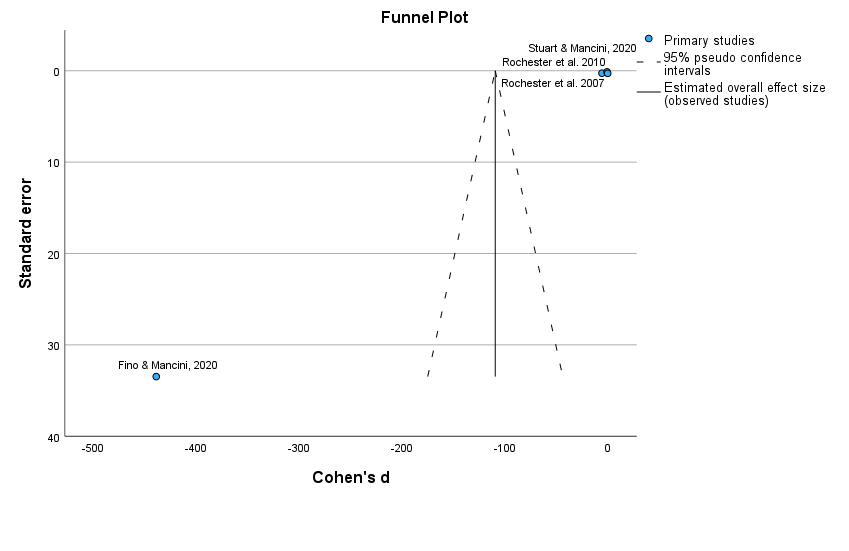


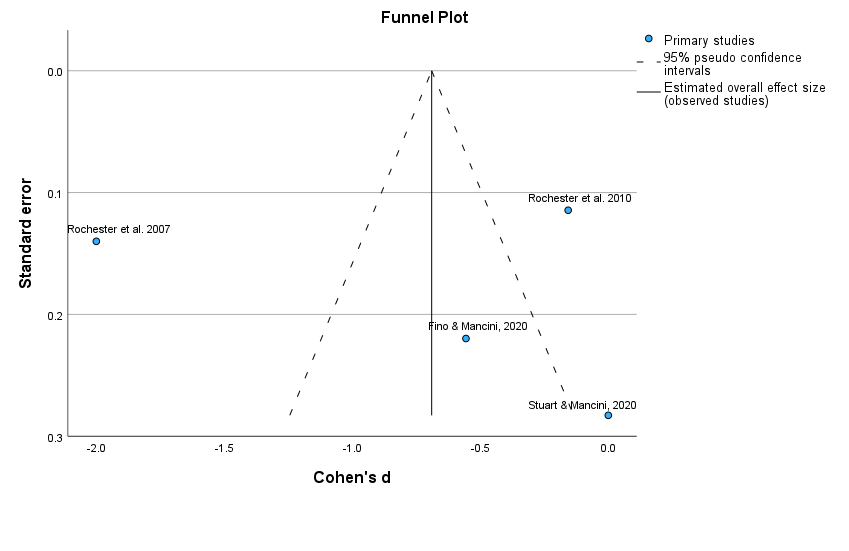


1. Step length


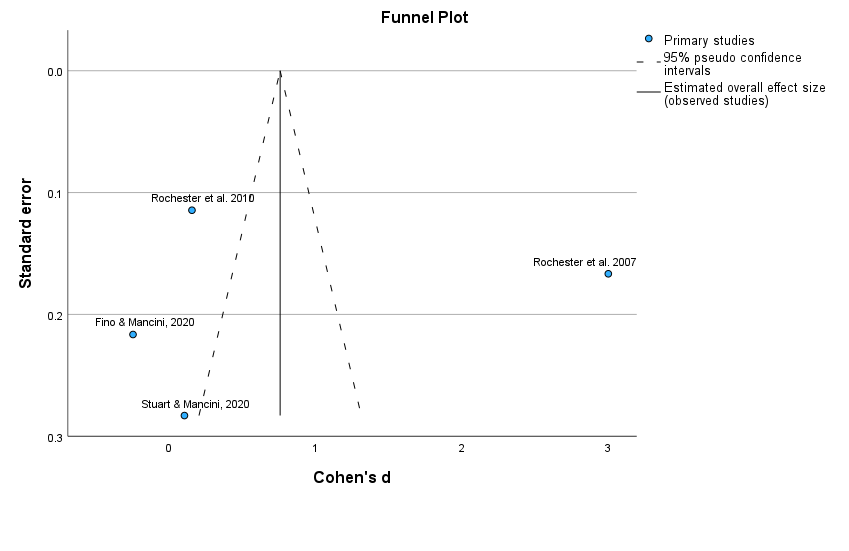


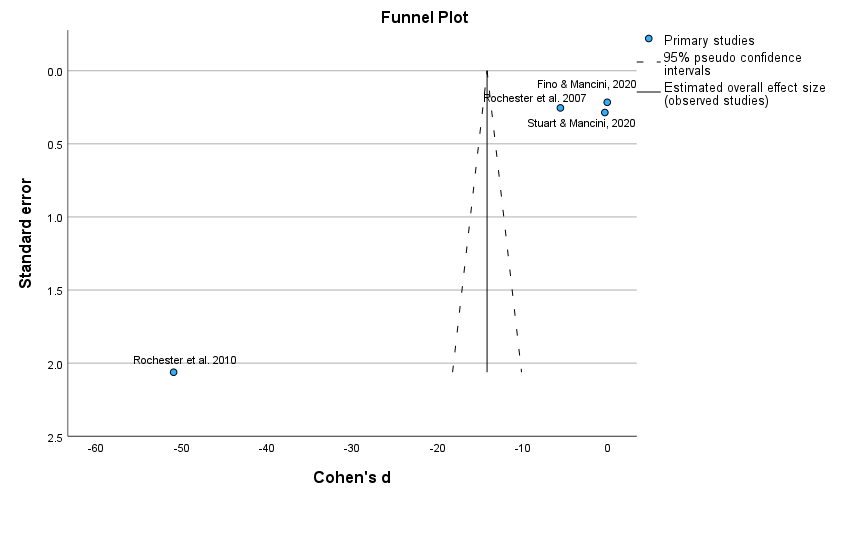


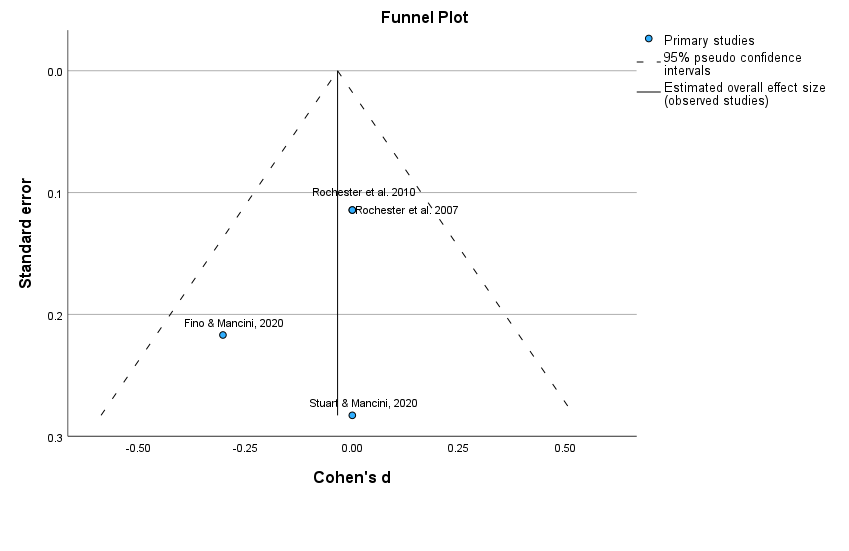


## **Figure A.** Funnel plot of meta-analysis on the effect of tactile cueing on dual task, dual task cost, and single task conditions, respectively, for i) walking speed and ii) step length.
